# Notch signalling in T cells: bridging tumour immunity and intratumoral cellular crosstalk

**DOI:** 10.3389/fimmu.2025.1659614

**Published:** 2025-10-02

**Authors:** Jasmine Sultana, Pritha Roy Choudhury, Saurav Bera, Mohona Chakravarti, Aishwarya Guha, Prodipto Das, Juhina Das, Gayatri S Iyer, Anirban Sarkar, Sukanya Dhar, Nilanjan Ganguly, Rathindranath Baral, Anamika Bose, Saptak Banerjee

**Affiliations:** ^1^ Department of Immunoregulation and Immunodiagnostics, Chittaranjan National Cancer Institute (CNCI), Kolkata, India; ^2^ Surgery Branch, Center for Cancer Research, National Cancer Institute, Bethesda, MD, United States; ^3^ KIIT School of Biotechnology, Kalinga Institute of Industrial Technology, Bhubaneswar, Odisha, India; ^4^ Department of Pharmaceutical Technology (Biotechnology), National Institute of Pharmaceutical Education and Research (NIPER), SAS Nagar, Punjab, India

**Keywords:** notch, T cells, macrophages, dendritic cells, myeloid derived suppressor cells, B cells, targeted-therapy

## Abstract

**Background:**

Notch receptor–ligand interaction is ubiquitous and fundamental for coordinating cellular differentiation and determining cell fate for the development of various tissues and organs. Aberrant mutations in the Notch cascade result in various pathophysiological disorders, including cancer. Diverse aspects of carcinogenesis regulated by Notch include the shaping of anti-tumour T-cell immunity through antigen-presenting cell (APC)–T cell interaction and effector functions.

**Chief content:**

Notch depends on juxtacrine and paracrine signalling to influence intercellular communications in the tumour microenvironment. Several preclinical and clinical studies have revealed Notch as a bi-effector molecule, which has a differential effect depending on the immune contexture of the tumour microenvironment. The Notch cascade serves as an effective therapeutic target in preventing off-target cell death and promoting tumour-specific T-cell priming.

**Conclusion:**

This review revolves around Notch crosstalk with respect to the interaction between T-cell populations and other intratumoral cellular components, including professional antigen-presenting cells like dendritic cells, macrophages, B cells, immunosuppressive myeloid-derived suppressor cells, and cancer stem cells. It also summarizes the impact of targeting Notch signalling within intratumoral T cells in combination with traditional oncotherapies.

## Introduction

The immune modulatory functionality of Notch signalling depends on both the involved cell and environmental cues; it becomes further multifaceted within the tumour microenvironment (TME). Tumour intrinsic crosstalk between immune cells, stromal cells, and malignant cells is regulated via Notch-induced reciprocal signalling (juxtacrine and paracrine) networks. Tumour cells often hijack these pathways and reroute them for their own benefit. The Notch signalling pathway plays both oncogenic and tumour-suppressor roles depending upon the type of malignancy. Notch is considered to be an oncogene in multiple malignancies of lymphoid origin (splenic marginal zone lymphoma, T-cell acute lymphoblastic leukaemia, and B-cell chronic lymphocytic leukaemia) (1–3). On the contrary, it is denoted as a tumour suppressor in myeloid malignancies (4, 5). Therefore, to ascertain and design anti-Notch therapeutics, an extensive look at the TME is required. Thus, the aim of this review was to illustrate this labyrinthine Notch signalling network within the TME with special emphasis on T-cell fate, their regulatory mechanisms, and therapeutic applications.

## The Notch pathway: receptors, ligands, and downstream signalling

Almost a century ago, Thomas Hunt Morgan and colleagues first discovered the Notch protein in a strain of fruit flies (*Drosophila melanogaster*) where the haplo-insufficiency of the Notch locus resulted in notched wings [R-Morgan, 1917]. This evolutionarily conserved signalling molecule influences differentiation, morphogenesis, proliferation, apoptosis, and cell fate determination (6). A summary of the Notch receptor–ligand interaction controlling immune cell fate is shown in **Table 1**.

**Table 1:** Notch signalling in the developmental fate of different immune cells.

Immune cells	Notch receptor	Notch ligand	Developmental stage modulated by the specific immune cell type	References
NK cells	Notch1 and 3	DLL1, Jagged1 and 2	Development of functional NK cells from hematopoietic progenitor cells.	(15–17)
	Notch1 and 2	DLL1 (decidual NK cells) and DLL4 (both peripheral blood and decidual NK cells)	Induce cytotoxicity of NK cells by upregulating IFN-γ secretion.	(18)
	Notch2	Jagged2	DC-mediated cytotoxicity of NK cells is increased.	(19)
	Notch1	DLL1 and 4	Development of NK cells from pro-B cells or common progenitor lymphoid cells.	(20)
Macrophages	Notch1	DLL1 and Jagged1	M0 macrophages are polarized into M1 macrophages with proinflammatory functionalities by inhibiting SIRPα.	(21–23)
	Notch1 and 3	DLL1 and 4	NF-κB-dependent proinflammatory gene expression is upregulated in TLR4-activated macrophages.	(24)
B cells	Notch1	DLL1 and Jagged1	Development of plasma cells occurs, and antibody production of the cells is also increased.	(25, 26)
	Notch2	DLL1	Upon BCR stimulation, follicular B cells are developed into marginal zone B cells.	(27, 28)
	Notch1	DLL1 and 4	Development of B cells from pro-B cells or common progenitor lymphoid cells is inhibited.	(20)
T cells	Notch3	DLL1	Naïve CD4^+^ cells developed into Th1 T helper cells.	(29)
	N/A	DLL1 and 4	DC-mediated signalling inhibited Th2 cell development.	(30)
	Notch1	DLL4	Synergistically with TGF-β, it induces Treg cell differentiation by inducing Foxp3 signalling.	(31, 32)

NK, natural killer cells; DC, dendritic cell; TGF-β, transforming growth factor beta; BCR, B cell receptor.

The Notch signalling is activated when any one of the four Notch receptors (Notch1–4) binds to their ligands, Jagged1/2 or Delta-like1–3. In humans, the genes of four Notch receptors are positioned on chromosomes 9, 1, 19, and 6 (7). In particular, Notch is a heterodimeric receptor comprising the Notch extracellular domain (NED), a transmembrane domain, and a Notch intracellular cytoplasmic domain (NICD). NICD harbours several conserved elements, such as protein–protein interaction, transactivation domains, and nuclear localization signals (NLSs) (8). The N-terminal region of Notch possesses a negative regulatory region (NRR) with 36 epidermal growth factor (EGF)-like repeats. The NRR contains 12 cysteine-rich repeats and a site that helps form heterodimers for S2 cleavage (9). Both Notch ligands and receptors are transmembrane proteins involved in cell-to-cell interaction. The Notch receptor interacts with an association region for the recombination signal binding protein for immunoglobulin kappa J region (RBPJ). The RBPJ association module has seven ankyrin repeats (ANKs) and helps in interaction with the nuclear transcription factors (10).

Proline/glutamic acid/serine/threonine-rich motifs (PEST domains) are located in the terminal parts of the intracellular region; these contain signals for degradation and hence are crucial for the stability of NICD (11). Mammalian Notch2–4 have structures resembling Notch1, differing solely in the number of EGF-like repeats, the extent of glycosylation of the EGF-like repeats, and the span of PEST domains. The expression level of Notch receptors on the cell membrane is regulated by endocytosis. During activation, the Notch receptors are first targeted by an ADAM-family metalloprotease (ADAM 10), which targets the NED. It is succeeded by a series of proteolytic cleavages and mediated by the γ-secretase complex and the translocation of NICD to the nucleus to regulate cellular gene expression (12). Once trafficked into the nucleus, NICD heterodimerizes with RBPJ, a DNA-binding transcription factor, and gives rise to a short-lived nuclear transcription complex, which is supported by co-activators MAML belonging to the Mastermind-like family (MAML1–3) (13, 14).

**Figure 1:**
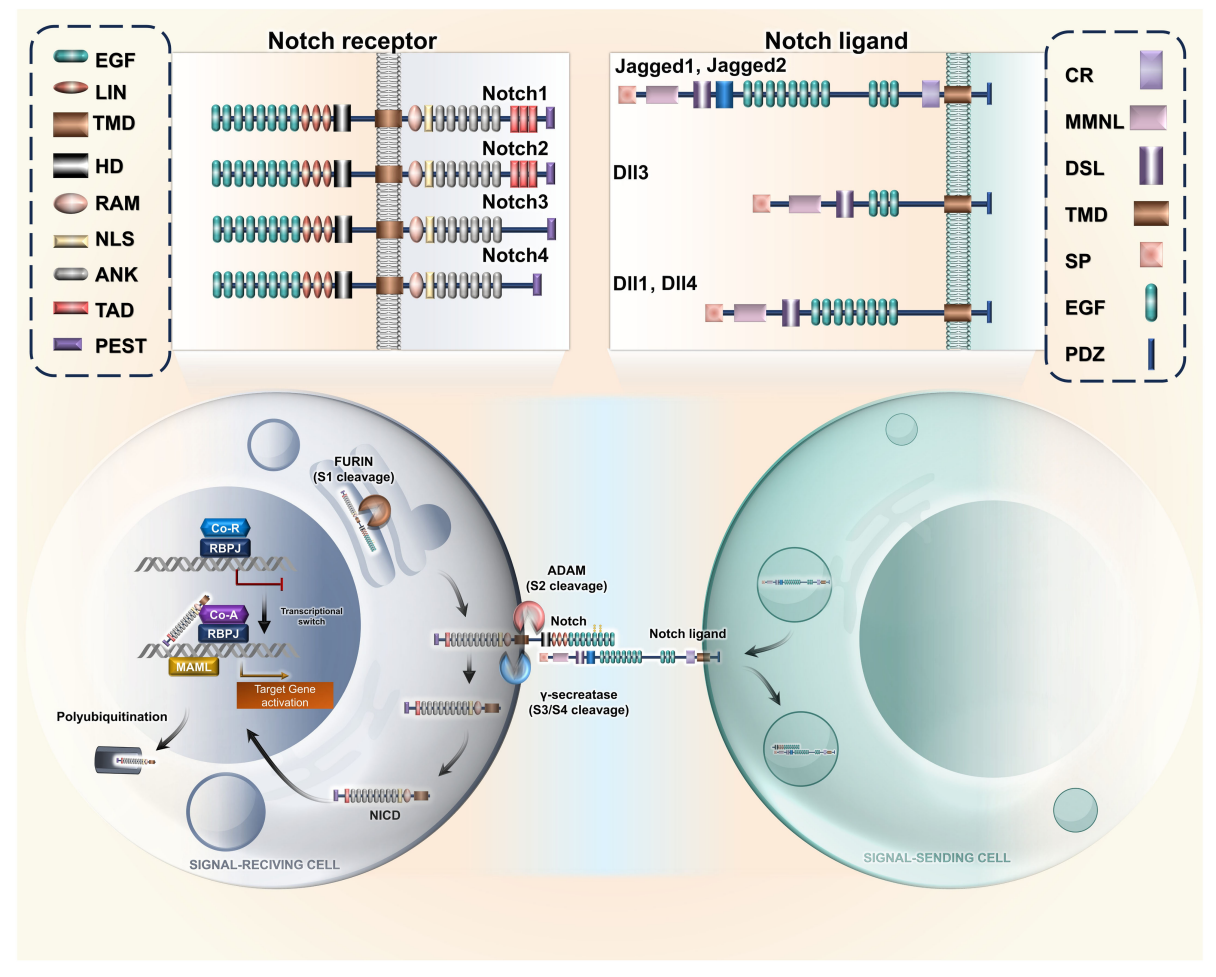
Notch receptor–ligand interaction. Representative illustration of interaction between the varied Notch ligands with their corresponding receptors, resulting in cleavage of intracellular domain due to activity of ADAM proteases, resulting in nuclear translocation of NICD. The acronyms or abbreviations have been added to the abbreviation list. EGF, epidermal growth factor; TMD, transmembrane domain; HD, heterodimerization domain; LIN, lineage defective; RAM, RBP-Jκ-Associated Module; NLS, nuclear localization signal; ANK, ankyrin repeat; TAD, transactivation domain; PEST, proline glutamine serine threonine; DLL, Delta-like ligand; CR, cysteine-rich; MMNL, module at the N−terminus of Notch ligands; DSL, Delta/Serrate/LAG-2; SP, signal peptide; PDZ, protein–protein interaction module; MAML, Mastermind-like; CoA, coactivator; CoR, corepressor; RBPJ, recombination signal binding protein for immunoglobulin kappa J region; ADAM, A Disintegrin And Metalloprotease; NICD, Notch intracellular domain.

This leads to the activation of downstream genes like hes (Hairy enhancer of split) and hey (Hairy related). In spite of similarities in the structure, the four Notch ligands have differential effects on various tissue types, which allows high plasticity in the regulation of gene expression (10). Cellular differentiation and communication are mostly regulated by DLL1. DLL4 has been noted to promote metastasis through VEGF secretion (33). JAG1 is reported to promote angiogenesis. However, JAG2 enhances cell proliferation (34, 35). The receptor–ligand interaction Notch is exhibited in **Figure 1**.

## Notch signalling regulates T-cell fate

Studies pertaining to the immune system have proven that Notch signalling influences several steps of T-cell development in central and peripheral lymphoid organs. It is identified to have a monumental role in T-cell development, differentiation, and maturation. Prior to T-cell receptor (TCR) ligation, Notch receptors are densely expressed in naïve T cells. Both thymopoiesis and T-cell maturation are spatially organized in the thymus (36). The lymphoid progenitors come into contact with overexpressed Notch ligands in the thymic cortex, and they face Notch-associated cues, which determine T-cell lineage commitment. Notch signalling continues till the pre-TCR checkpoint, beyond which it is downregulated (37). In double-positive (CD4^+^CD8^+^) thymocytes, the expression of Notch decreases to avoid the probable intervention in positive and negative selection. TCR stimulation causes CD4^+^ T cells to differentiate into several subsets. Inversely, JAG1 or JAG2 ligands are considered more important for the Th2 subtype (38). After antigen exposure, CD8^+^ T cells differentiate into either short-lived effector cells (SLECs) or memory precursor effector cells (MPECs). Notch signalling regulates the differentiation of SLECs following dendritic cell (DC) immunization. The SLEC population dwindles in the absence of Notch signalling. However, Notch is dispensable in the MPEC differentiation process (39).

Tumorigenesis manifests as immune suppression and hinders intrathymic maturation or differentiation of T cells. It mediates a loss in the pool of CD4^+^CD8^+^ immature thymocytes and a steady increase in CD4^−^CD8^−^ progenitors of T cells. A study by Guha and colleagues (40) elaborated that the progression of malignancy inhibits the modulation of Lineage^−^Thy1.2^+^CD25^+^CD44^+^c-Kit low DN2b to Lineage^−^Thy1.2^+^CD25^+^CD44^−^c-Kit^−^DN3 during T-cell maturation. It was also reported that there was a downregulation of Notch1 and downstream targets like Ikaros, irf8, and pu.1, which direct the transition of DN2a towards dendritic cell lineage commitment. Corresponding Notch ligands like DLL1 or DLL4, on CD4^+^ T-cell activation, skew them to the Th1 fate. Moreover, it was also reported that the interaction of interleukin-10 with IL-10 receptor high DN2 thymocytes further drives DC lineage commitment by STAT3 phosphorylation (40).

## Notch signalling regulates T-cell functionality

The activation of Notch1/2 in CD8^+^ T cells boosts anti-tumour response, increasing IFN-γ production and reducing tumour load (41). DLL1 (human homolog of Notch ligand) expression on DCs and bone marrow cells magnifies T-cell infiltration in tumours. It also increases IFN-γ production and mouse survivability and decreases tumour burden (42). Moreover, Notch-dependent increased functionality of antigen-specific CD8^+^ tumour-infiltrating lymphocytes (TILs) was also reported. Primarily, the clustered form of DLL1 (c-DLL1) binds and activates Notch (1–4), resulting in Notch-mediated/targeted gene expression, increasing the tumour infiltration of antigen-specific T cells and the regression of tumour (43). By inhibiting or knocking down Notch expression (mainly Notch2), it was found that the differentiation and promotion of the cytolytic function of murine T cells were prohibited both *in vitro* and *in vivo*. These observations specified that Notch2 is decisive for the anti-tumour reaction of cytotoxic T lymphocyte (CTL) cells. The three complexes comprising phosphorylated CREB1, activated NICD, and transcriptional co-activator p300 enhance Notch transcription by binding to the promoter of the granzyme B gene, thus enhancing its transcription (44). However, many counteractive effects of Notch signalling in intratumoral T cells were also evident. In colorectal carcinoma, Notch cascades inhibit proinflammatory cytokine (IFN-γ, TNFα, IL-1β, IL-6, and IL-8)-dependent non-cytolytic anti-tumour function of CD8^+^ T cells. Apart from increasing the effector immune response of CD8^+^ T cells, Notch signalling can also hasten T-cell exhaustion. The inhibition of Notch signalling led to a significant decrease in programmed cell death protein 1 (PD-1) expression of CD8^+^ T cells. The activation complex of Notch signalling was observed to accelerate PD-1 transcription in CD8^+^ T cells by binding to its promoter. In colorectal carcinoma, it was observed that the Notch expression elevated the PD-1 expression in CD8^+^ T cells (45). The multifaceted role of Notch signalling in T-cell activity is shown in **Table 2**.

**Table 2:** The diverse effects of Notch on T cells in different types of cancer.

Types of malignancy	Modulation of Notch expression	Effect on T cells	Reference
Lung cancer	Overexpression of Notch1 in cancer cells.	Increased glycolysis in cancer cells via Notch1/TAZ axis. It leads to excess lactate production, which inhibits the lactate release of T cells. Due to the intracellular lactate accumulation, T-cell proliferation and cytokine release are abrogated, resulting in reduced cytotoxic T-cell function.	(47)
	Multivalent DLL1–Notch1 interaction.	Increased anti-tumour T-cell response and enhanced efficacy of epidermal growth factor receptor targeted therapy.	(48)
Ovarian cancer	Reduced Notch expression in T cells via restriction of EZH2 by reducing aerobic glycolysis.	Reduced effector T-cell survival and cytokine release. Altogether, T cell-mediated anti-tumour activity is attenuated.	(49)
EG7 thymoma	Notch signalling is stimulated.	Notch2 signalling directly regulates transcription of genes encoding the crucial CTL effector molecules granzyme B and perforin.	(44, 50)
Leukaemia	Increased expression of Notch ligand is observed in mouse model.	DLL4 shows a great capacity to promote the generation of T helper (Th)1 and Th17 CD4^+^ T cells.	(51)
Hepatocellular carcinoma	Blockade in nuclear translocation of the intracellular motif of Notch2 by overexpression of *1810011o10Rik* (*Tcim*)	The cytotoxic effect of CD8^+^ T cells was inhibited.	(52)

CTL, cytotoxic T lymphocyte.

## Notch signalling in T-cell dysfunction

The development and growth of cancer generate dysfunctional or exhausted CD8^+^ T cells, which assist immunotolerance and correspond with tumour metastasis. This results in failure of functional peripheral blood CD8^+^ T cells, which is revealed by depleting the cytotoxic effects (for example, by perforin/granzyme pathway and Fas/FasL pathway). This involves an increase in the expression of inhibitory immune receptors or regulatory molecules like PD-1, cytotoxic T lymphocyte-associated protein-4 (CTLA-4), and T-cell immunoglobulin and mucin domain-containing-3 (TIM-3), thus creating a tolerogenic environment. The suppression of the Notch signalling pathway in CD8^+^ T cells from prostate cancer (PCa) patients results in the decreased expression of downstream signalling molecules Hes1 and Hes5. Hes1 maintains the proliferation of progenitor cells, and it is an essential effector of the Notch signalling pathway (46). Notch activation enhances the direct cell killing capability of CD8^+^ T cells, fostering the expression of cytotoxic molecules while diminishing inhibitory immune regulatory molecules.

**Figure 2:**
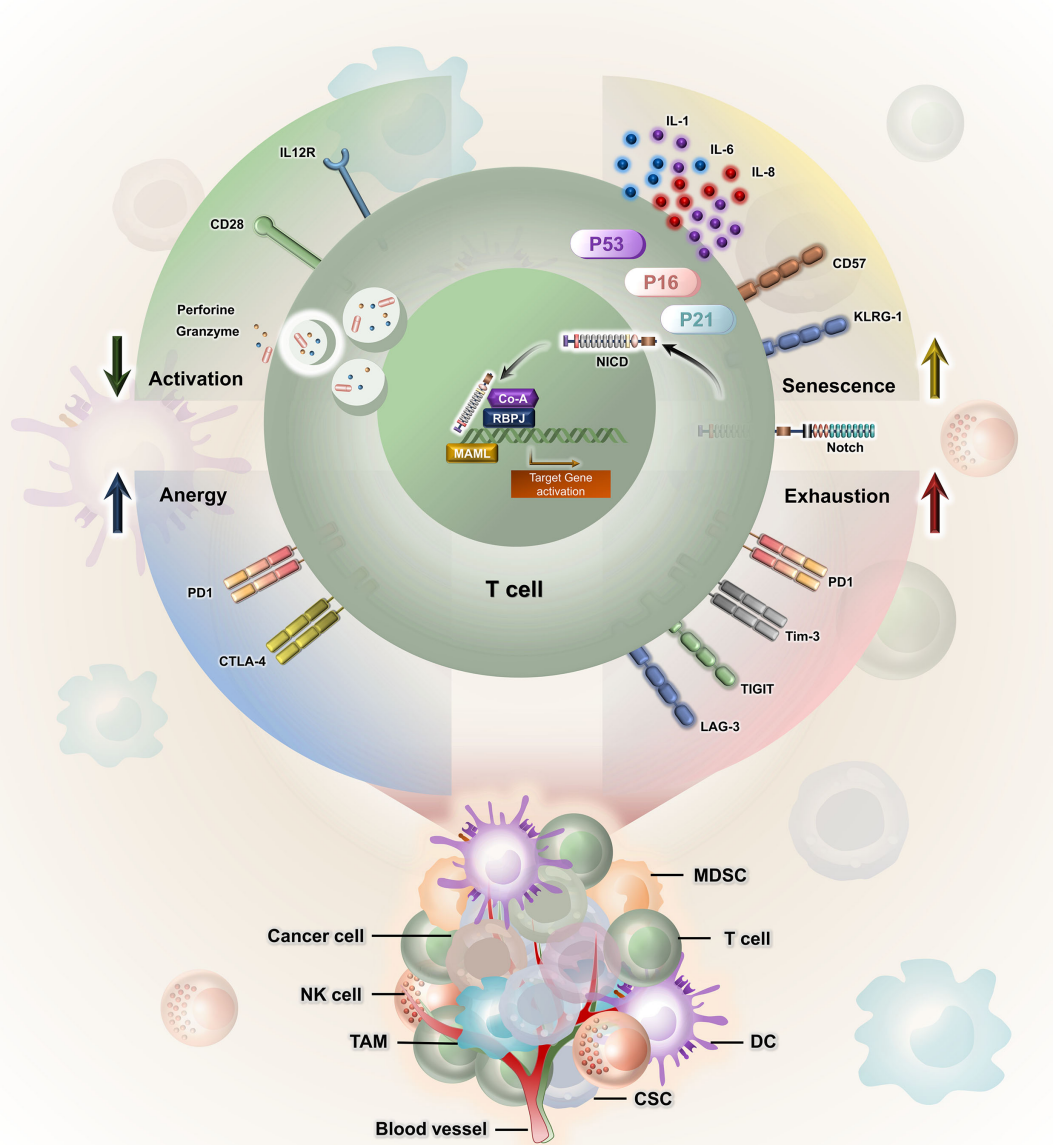
Notch signalling has bi-effector functions in T cell-mediated anti-tumour immunity. Notch receptor–ligand interaction controls senescence, exhaustion, anergy, and activation of T cells by regulating the expression of various associated molecules like KLRG1, PD-1, Tim3, LAG-3, CTLA-4, perforin, and granzyme. CSC, cancer stem cell; MDSC, myeloid-derived suppressor cell; DC, dendritic cell; TAM, tumour-associated macrophage; PD-1 1, programmed cell death protein 1; CTLA4, cytotoxic T-cell lymphocyte-associated protein 4; KLRG, killer cell lectin-like receptor subfamily G member; TIGIT, T-cell immunoreceptor with Ig and ITIM domains; TIM3, T-cell immunoglobulin and mucin domain 3.

It was also observed that due to the distinct patterns of gene activity observed in Notch1 and Notch2 within colorectal carcinoma samples, Notch signalling plays a role in the detriment of CD8^+^ T-cell functions during the development of colorectal carcinoma. The inhibition of Notch signalling reduces PD-1 expression on CD8^+^ T cells in patients with colorectal carcinoma; however, the frequency of CTLA-4^+^ cells within CD8^+^ T cells did not have a significant change in either peripheral blood (healthy individuals and patients) or colorectal carcinoma specimens (45). Notch signalling plays a significant role in T-cell senescence in cancer, influencing various aspects of T-cell function and fate. Dysregulated Notch signalling is often characterized by increased Notch receptor activation and has been linked to the induction of senescence-associated changes in T cells. Senescence-associated secretory phenotype (SASP) is illustrated by the presence of various proinflammatory cytokines, including IL-1, IL-6, and IL-8. The activated Notch1 intracellular domain (N1ICD) and the conventional Notch1 target, HES1, showed a transient increase during the shift towards senescence. Nevertheless, as senescence reached its full extent, both the cleaved Notch1 intracellular domain and HES1 returned to levels close to their baseline. It was observed that there was a temporary increase in transforming growth factor beta (TGF-β) ligands during both Replicative-Induced Senescence (RIS) and DNA Damage-Induced Senescence (DDIS). This trend shows the expression pattern of N1ICD. These findings suggest that there is a temporal correlation between Notch signalling and the concurrent induction of TGF-β and proinflammatory cytokines throughout the senescence process (53). Understanding the tangled interplay between Notch signalling and T-cell anergy, exhaustion, and senescence is crucial for unfolding the molecular mechanisms and the therapeutic targets for age-related immune dysfunction associated with aberrant T-cell responses. The impact of Notch signalling in T cell-mediated anti-tumour immunity is showcased in **Figure 2**.

## Notch as a bi-effector molecule in the tumour niche

Several lines of evidence have suggested that Notch can also act as a tumour suppressor by shaping the tumour immune microenvironment. It modifies the immune milieu and stromal interactions. For example, it was demonstrated by Demehri et al. that Notch deletion alters cytokine secretion and interrupts epidermal homeostasis by promoting an inflammatory TME that advances tumour development (54–56). Parmigiani et al. reported that with Notch loss in brain tumour, an immune-suppressive environment was created that ultimately generated conditions favouring tumour progression (57). This highlights that Notch both functions as an oncogenic driver and exerts tumour-suppressive effects through the regulation of the TME.

The aberrant activation of Notch results in the initiation and progression of tumours. In colorectal cancer (CRC), Notch signalling controls the immune contexture and tumour cell morphology, promoting immune evasion and cancer progression (58). In breast cancer cells, Notch binds to JAG1 to secrete macrophage-polarizing cytokines like IL-β or CCL2, resulting in M2-type macrophage recruitment, producing TGF-β cues to tumour cells. Crosstalks such as these may be responsible for drug resistance, which needs further validation (59).

## Involvement of Notch signalling in CSC T-cell interaction

**Figure 3:**
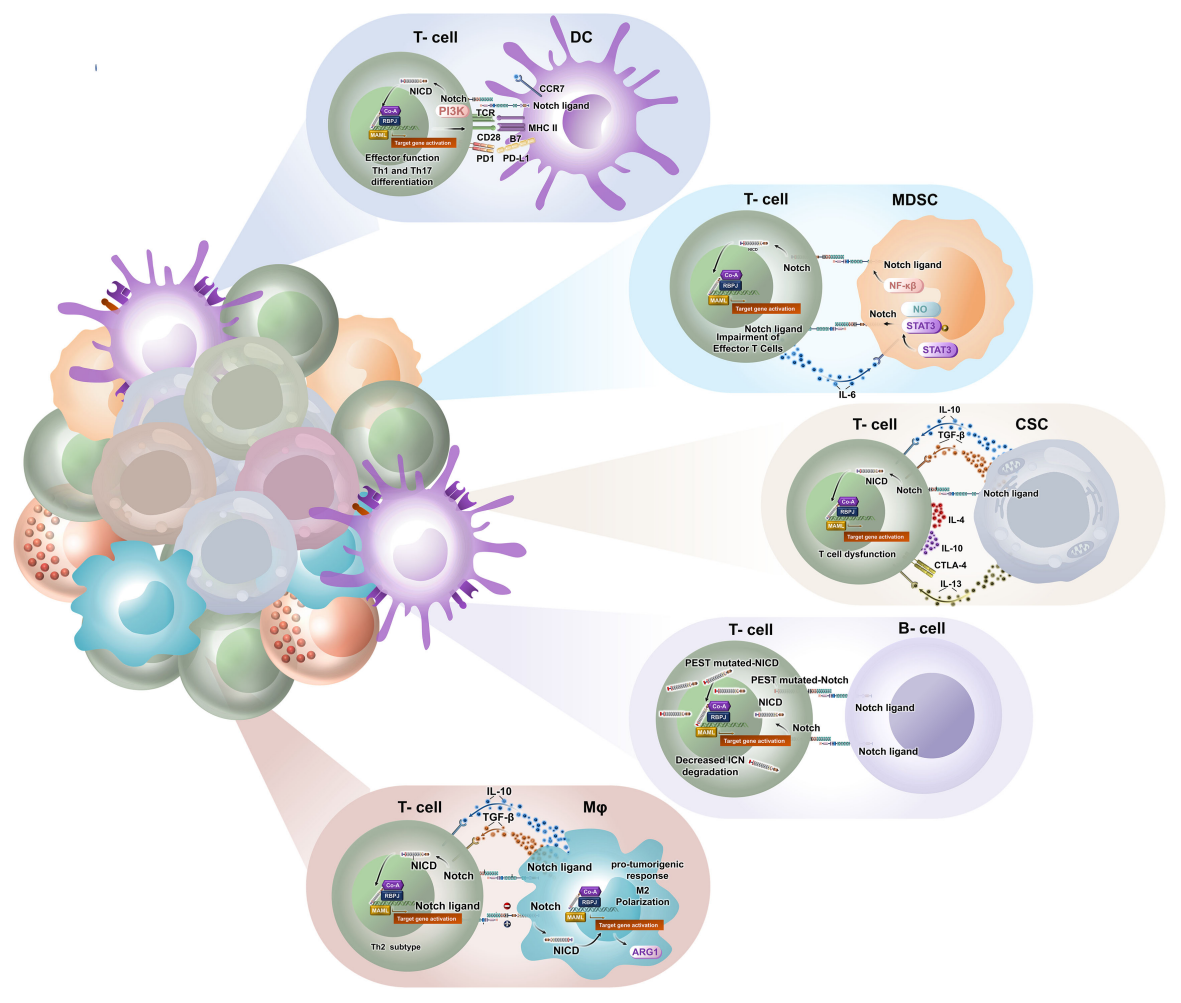
Notch signalling mediates bi-directional crosstalk between T cells and other intratumoral components to modulate tumour-immune niche. Notch promotes pro-tumorigenic activity by macrophage switching towards M2 phenotype through regulated secretion of cytokines like IL-10 and TGF-β. Notch signalling is also significant in antigen presentation and intratumoral infiltration of DCs. It directs Th1 and Th17 effector functions against tumour antigen. MDSCs hinder T cell-mediated cytotoxicity through NF-κB or Notch-Stat3-NO cascade. It also promotes intratumoral trafficking of monocytic suppressor cells. Notch upregulation along with TGF-β, IL-10, and IL-13 secretion by CSCs results in T-cell dysfunction. Such CSCs stimulate CTLA-4 expression on effector T cells. There is a huge lacuna in understanding the impact of Notch on B cells in cancer. TGF-β, transforming growth factor beta; DCs, dendritic cells; MDSCs, myeloid-derived suppressor cells; CSCs, cancer stem cells.

In addition to driving the growth of tumours through mesenchymal transition, cancer stem cells (CSCs) play a role in fostering immune evasion within the TME, allowing them to effectively evade the anti-tumour immune responses. Additionally, CSCs with the capacity to generate immunosuppressive molecules, like TGF-β, IL-10, and IL-13, contribute to immune dysfunctions (60). Notch1 plays an important role in governing luminal estrogen receptor (ER^+^) breast cancer and breast cancer stem cells (BCSCs). Interestingly, the levels showed an opposite relationship with the more aggressive triple-negative or basal-like BCSCs and the presence of infiltrating Foxp3^+^ Tregs. These Tregs produced inhibitory cytokines like IL-4 and IL-10, and they also expressed CTLA-4. Enhanced survival in various types of cancer cases was associated with elevated T-cell counts, particularly in activated CD8^+^ CTLs and Th1 cells. Studies have indicated that there is a direct role in regulating gene expression specific to CTLs, including granzyme B, played by both Notch1 and Notch2. Notably, CD8^+^ T cells lacking Notch2, but not those lacking Notch1, demonstrated an inability to proliferate and effectively inhibit tumour growth in mice (61). The Notch signalling-mediated crosstalk between T cells and other intratumoral components is depicted in **Figure 3**.

## Notch interplay in intratumoral APC–T cell interactions

Naïve T cells receive signals from antigen-presenting cells (APCs) such as DCs, macrophages, and B cells via the release of several cytokines, which have a significant role in both CD4^+^ and CD8^+^ T-cell differentiation.

## Notch promotes pro-tumorigenic activity in TAMs

In tumour-resident macrophages, the deletion of Rbpj limited the activity of cytotoxic T cells in B16 melanoma, and Notch activation improved CD8^+^ T-cell infiltration in Lewis lung carcinoma (LLC) (21, 62). Contrary to the previous observations, the activation of Notch in tumour-associated macrophages (TAMs) elicited pro-tumorigenic response in breast cancer models, including M2 polarization and hindrance of T-cell activity (63). *In vivo* experiments conducted in a Triple-negative Breast Cancer (TNBC) mouse model reported that intratumoral macrophages, which were educated by tumour-derived Jagged1, inhibited the proliferation and cytotoxicity of T cells via upregulated soluble molecules like CD93 and CD14 (64). A recent study also reported that activated Notch signalling in TAMs led to the upregulated expression of arginase 1, the key driver of immunosuppression, in an engineered mouse model of pancreatic cancer (65). Here, a combinatorial therapy of PD-1 blockade supported by Notch inhibition led to improved intratumoral T-cell infiltration, restriction of tumour size, and tumour cell apoptosis. Contemporary research has also suggested that Notch regulates the switching of macrophage phenotypes within the tumour microenvironment. Notch-deficient macrophages have phenotypes of M2 macrophages with a complex surface marker profile that skews naïve CD4^+^ T cells to produce Th2 subtypes (62). This was further evidenced in osteosarcoma, where the expression of Th2 cytokines like IL-10 and TGF-β was elevated in Notch1 knockout mice compared to control mice. In contrast, the expression of Th1 cytokines like IL-6, TNFα, and IL-1β was dampened in Notch1 knockout mice (66). Hence, it is suggested that Notch signalling in macrophages regulates phenotype switching and promotes tumour advancement.

## Notch stimulates intratumoral DC–T cell interplay

Notch has been elucidated as a prime signalling pathway in DC–T cell interaction for inducing the effector functions of CD8^+^ T cells. Studies on tumour-infiltrating immune cells in pancreatic and lung cancers have revealed an association between the expression of Notch ligands by tissue-resident DCs and the cytolytic activity of T cells expressing Notch receptors and PD-1. The expression of PD-1 is controlled by several cell-to-cell interactions, including Notch-driven transcriptional control of the PDCD-1 gene. Recent data on murine lung cancer suggest that mice generated with the deletion of Notch ligand DLL1 in the CD11c lineage promoted tumour growth and increased PD-1 expression on CD8^+^ T cells, which led to the inhibition of antitumoral T-cell function and long-term T-cell memory responses (52). Intratumoral T-cell infiltration was augmented in mice with the expression of DLL1 on bone marrow and DCs, thereby reducing tumour growth and prolonging mouse survival (53, 60). In mouse bladder cancer, DC subsets with upregulated DLL4 expression during exposure to low doses of antigen have been reported to promote phosphatidylinositol 3-OH kinase (PI3K)-mediated TCR–CD28 interaction, thereby potentiating CD4^+^ T cells to evoke elevated anti-tumour effect (67). A similar subset of DLL4^+^ DC population was found in human peripheral blood with inflammatory conditions and was found to aid Th1 and Th17 differentiation (68). Adoptive transfer of Notch-primed DCs restricted the progression of inflammation-associated colorectal carcinogenesis. Notch2 signalling mediated the upregulation of CCR7, promoting DC migration and cross-presentation to effector T cells, which was supported by the fact that RBPJ knockdown in murine DCs showed unrestricted tumour growth *in vivo* due to poor DC infiltration and subsequent antigen presentation (69). Hence, Notch activation aids DC-mediated T-cell functionality.

## Notch in intratumoral MDSC–T cell crosstalk in cancer

Tumours promote the process of immunosuppression and evasion of immunosurveillance by several kinds of suppressor cells, including myeloid-derived suppressor cells (MDSCs), which are represented by a heterogeneous population of immature myeloid cells. Sierra et al. (2015) investigated the importance of Notch1 and Notch2 expression in intratumoral CD8^+^ T cells of Lewis lung carcinoma. Experimental knockout of Notch1 and Notch2 led to decreased production of IFN-γ and restricted the proliferation and infiltration of activated CD8^+^ T cells (70). MDSCs hindered the expression of Notch1 and Notch2 on T cells via a nitric oxide-dependent cascade. In the same study, it was shown that the transgenic expression of N1ICD in antigen-specific effector T cells had no impact on T-cell activation or proliferation but resulted in increased cytotoxicity and induced a T-cell phenotype with central memory. These effector T cells were resistant to MDSC-mediated tolerogenicity. In a similar collaborative study, they reported that tumour-dwelling MDSCs upregulated the expression of Notch ligand Jagged1 via the NF-κB pathway. In this case, significant prognosis was observed in mouse models of 3LL lung carcinoma and EG-7 lymphoma when treated with anti-Jagged1/2 antibody. The therapeutic response was based on increased CD8 functionality (71). Anti-Jagged therapy in EG-7 tumour intensified the impact of anti-ovalbumin adoptive T-cell therapy (ACT) (72). A study on breast cancer patients suggested that MDSCs augmented tumour progression by hampering the production of effector T-cell cytolytic enzymes like granzyme, perforin, and IFN-γ, as well as by enhancing breast cancer cell stem-like properties. Further research revealed that the mechanism pertained to a crosstalk between STAT3 and Notch pathways. It resulted in the induction of MDSCs to cause IL-6-dependent phosphorylation of STAT3, which led to the activation of Notch via the nitric oxide pathway (73). In a central nervous system cancer model, after oncolytic herpes simplex virus-based therapy, γ-secretase inhibitor (GSI)-mediated Notch blockade in myeloid cells reduced CCL2 secretion, thereby preventing the infiltration of immunosuppressive monocytic MDSCs to infect tumour sites and promoting T cell-mediated killing (74).

## Notch dysregulation in T-cell malignancies

According to prior reports, Notch mutation drives T-cell acute lymphocytic leukaemia (T-ALL). In T-cell malignancies, specifically T-ALL, Notch1 is often mutated, influencing leukemic shift by supporting proliferation and hindering differentiation. Persistent Notch activation results in unregulated proliferation, constrains differentiation, and improves the survival of leukemic cells through the transcriptional increase of oncogenic targets such as MYC, HES1, and IL7R. Frameshift, nonsense, or alternative splicing mutations, which affect the PEST domain, are sometimes noted in the absence of mutations, disturbing other negative regulatory regions. PEST mutations are mostly observed in chronic lymphocytic leukaemia, mantle cell lymphoma, and peripheral T-cell lymphomas like adult T-cell leukaemia/lymphoma (75).

## Notch-mediated oncotherapy involving T cells

Given the importance of Notch in determining immune cell differentiation and consequently moulding the range and variety of immune responses in the TME, several clinicians and researchers have identified Notch as a potential target for therapy. Multiple preclinical studies have unravelled many T-cell therapeutic facets involving the Notch pathway. Some of them are discussed below.

### γ-Secretase inhibitors in T cell-mediated oncotherapy

A study conducted by Yu and colleagues (2018) on colorectal cancer indicated that the inhibition of the Notch cascade via *N*-[*N*-(3,5-difluorophenacetyl)-l-alanyl]-*S*-phenylglycine *t*-butyl ester (DAPT), which is a well-known GSI, resulted in improved cytotoxicity of CD8^+^ T cells infiltrating the TME. DAPT upregulates proinflammatory cytokine production comprising TNFα, IL-1β, IFN-γ, IL-6, and IL-8 from a tumour-infiltrating effector CD8^+^ T-cell population. DAPT treatment also resulted in the reduced expression of PD-1 exhaustion marker with little impact on cell proliferation. Therefore, Notch blockade improved cytolytic and cytokine-dependent effector functions of T cells in colorectal cancer (22). A recent study assessed the effect of γ-secretase inhibitors in T cell-dependent lysis of multiple myeloma (MM) cells. The study disclosed that in *ex vivo* co-culture setups of MM cells and T cells, GSIs reversed the inhibition of lysis of MM cells induced by soluble B-cell maturation antigen and improved the capacity of BCMAxCD3 bispecific antibodies to lyse MM cells obtained from patients. Furthermore, GSIs also increased T-cell cytotoxicity by increasing the expression of TNFα, CD107a, IL-2, and IFN-γ (76).

### Immune checkpoint blockade involving Notch activation

In small cell lung carcinoma (SCLC), the activation of Notch signalling confirmed good clinical prognosis in cohorts with relapsed SCLC receiving immune checkpoint blockade (ICB) therapy (82). In another study on SCLC, the suppression of lysine-specific demethylase 1a (LSD1) led to the activation of Notch signalling. Further investigation suggested that LSD1 inhibition by bomedemstat and consequent Notch activation evoked a better response to anti-PD-1 therapy (77). Contrastingly, in gastric cancer, Notch3 seemed to be inversely correlated with the biomarkers of ICB therapy, including gene expression profiling (GEP), tumour mutational burden (TMB), and innate anti-PD-1 resistance (IPRES) signature, suggesting its importance as a predictive biomarker (78). Combinatorial ICB therapy with GSI restricted tumour progression in triple-negative breast cancer (64). Recent transcriptomic data analysis showed that the increased expression of Notch1/4 enhanced relapse in ER-negative breast cancer. The expression patterns of three intratumoral cells, namely, MDSCs, TAMs, and cancer-associated fibroblasts (CAFs), are known to hinder intratumoral T-cell infiltration, which is essential for the determination of the T-cell exclusion score. In ICB therapy of ER-negative breast cancer, Notch1/4 was synergistically linked with T-cell exclusion score (79). Cytotoxicity-associated molecules like granzyme B, perforin 1, and exhaustion marker PD-1 were upregulated in colorectal carcinoma with Notch mutation (80).

### Notch in CAR-iTSCM oncotherapy

A strategy to overcome the scarcity of patient-derived T cells is to generate chimeric antigen receptor (CAR) T cells derived from induced pluripotent stem cells of cancer patients. The production of T cells from induced pluripotent stem cells (iPSCs) is directed by stromal cells that express DLL1 and activate the Notch pathway. Comparable methods have enabled the production of CAR T cells from hematopoietic stem cells; reprogramming factors are added for the induction of pluripotency and have been applied for the proliferation of tumour-specific human T cells. Although this approach facilitates the production of unlimited tumour-specific cytotoxic T lymphocytes, it often results in a low TCR repertoire.

To resolve this, investigators are presently investigating the use of T-stem cell memory (TSCM) cells in the adoptive transfer of T cells, exploiting their high *in vivo* persistence, self-renewing trait, and multipotency. Adoptive T-cell transfer of CAR T cells produced from TSCM cells initiates more effective anti-tumour responses than those produced from other T-cell subsets (81). Previous experiments conducted by Kondo et al. employed Notch signalling activation to produce induced T memory stem cell (iTSCM) of mouse or human origin. The key traits of TSCM that are also present in iTSCM cells include their ability to show rapid response to antigen re-stimulation and heightened self-renewal efficiency. Moreover, the iTSCM cells also showcase reduced expression of immune checkpoint markers like PD-1 and CTLA-4. The iTSCM cells are produced from intratumoral activated T lymphocytes (81).

### Notch inhibition with oncolytic virotherapy

Contemporary research projects the clinical use of oncolytic viruses, specifically herpes simplex virus, as an emerging therapy to kill cancer cells, sparing normal ones. Studies on glioblastoma have revealed that oncolytic herpes simplex virus (oHSV) infection significantly upregulates Notch signalling, stimulating TAMs to produce an increased number of CCL2 and IL-10 and promotes the accumulation of MDSCs in the TME. GSI-mediated Notch blocking improved CD8^+^ T cell-oriented anti-tumour memory response and helped in overcoming the immunosuppressive effect of oHSV therapy. Combinatorial oncolytic virotherapy administered alongside DAPT, GSI, or Notch antibodies showed decreased expression of PD-1 on effector T cells. The treatment of GL261N4-bearing mice receiving combinatorial therapy with depleting antibodies of CD4 and CD8 decreased their survival percentage, emphasizing the significance of tumour-specific T-cell memory response. In glioma mice having decreased Notch expression, cytotoxic T-cell secretions like IFN-γ and granzyme B were compromised (74). The previously described Notch-mediated T-cell oncotherapy is summarized (**Table 3**).

**Table 3:** Impact of Notch signalling in T cell-mediated oncotherapy.

Type of oncotherapy	Type of malignancy	Notch-mediated effect	Effect of malignancy progression	Reference
γ-Secretase inhibitors (GSIs)	Colorectal cancer	Notch downregulates proinflammatory cytokine production comprising TNFα, IL-1β, IFN-γ, IL-6, and IL-8 from tumour-infiltrating effector CD8^+^ T-cell population.	Notch blockade improved cytolytic and cytokine-dependent effector functions of T cells in colorectal cancer.	(22)
Immune checkpoint blockade (ICB)	SCLC	The inhibition of lysine-specific demethylase 1a (LSD1) led to the activation of Notch signalling. LSD1 suppression by bomedemstat and resulting Notch activation induced better response to anti-PD-1 therapy	Good clinical prognosis in cases of relapsed SCLC receiving ICB therapy.	(77, 82)
Oncolytic herpes simplex virus (oHSV)	Glioblastoma	Upregulation of Notch signalling stimulates TAMs to produce increased number of CCL2 and IL-10.	Decreased Notch expression resulted in compromised cytotoxic T-cell secretions like IFN-γ and granzyme B.	(74)
CAR-iTSCM cell therapy	Multiple myeloma	Notch1 signalling activation is required for increased CAR T-cell proliferation.	Reduced expression of CTLA4 and PD-1.	(81)

SCLC, small cell lung carcinoma; TAMs, tumour-associated macrophages; CAR, chimeric antigen receptor.

### Harnessing the versatility of Notch in T cell-based oncotherapy

In order to demonstrate the flexibility of the Notch receptor’s activation, here, we discuss synthetic Notch (SynNotch) receptors. SynNotch CAR T cells are an innovative strategy of engineered T cells that integrate SynNotch receptors and CARs on T cells to facilitate target specificity and control proliferation in onco-immunotherapy. SynNotch receptors repurpose the essential proteolytic domain of the innate Notch receptors. In SynNotch receptors, both the extracellular ligand-binding domain and the intracellular transcriptional domain of Notch are substituted with customized modules. Significantly, while the SynNotch system depends on the ligand-mediated proteolytic cleavage mechanism of Notch, it never activates the endogenous Notch signalling pathway. This modular design allows diverse responses upon the recognition of ligand and underscores the enormous potential of Notch-based systems in therapeutic applications (83).

Lim and colleagues (2020) generated the SynNotch system. SynNotch CAR T cells are engineered to recognize multiple tumour antigens (both intracellular and extracellular) and cause explicit tumour cell killing in a heavily heterogeneous cell population of solid tumours (84, 85). In contrast to traditional CAR T cells, which show an immediate response upon identifying the tumour antigen, SynNotch CAR T cells need a two-step activation procedure. The SynNotch receptor is synthesized to recognize an initial priming antigen of the tumour cells, thereby inducing the expression of a chimeric antigen receptor for the recognition of another tumour-associated antigen. This AND-gate logic works like a combinative antigen recognition system, which improves the efficacy of T cell-based oncotherapies. The strategy requires the availability of two separate antigens, one of which is recognized by the SynNotch receptor and the other by the CAR, ensuring that T cells undergo activation only within the context of the tumour microenvironment. This helps in overcoming off-target toxicity (86). These T cells upregulate transcription factors like T-bet, promoting Th1-mediated anti-tumour immunity. Furthermore, they produce T-cell proliferative and polarizing cytokines like IL-2 and IL-12. The SynNotch circuit prompts receptor-specific elimination of tumour cells and stimulates tumour killing via tumour necrosis factor-related apoptosis-inducing ligand (TRAIL). SynNotch diminishes the population of exhausted and anergic T cells and sustains an increased number of T cells in a naïve or memory state (87).

## Concluding perspectives

Notch signalling is indispensable for regulating the dynamics of intratumoral cellular interactions. In the tumour microenvironment, Notch acts as a dual-edged sword. Observational and clinical evidence suggests that the tumour microenvironment limits receptor and ligand-mediated Notch activation, subsequently repressing anti-tumour T-cell function. Notch convincingly promotes DC–T cell interaction, resulting in improved T-cell priming and subsequent reduction of tumour burden via T-cell cytotoxicity in animal models. The effect of Notch activation on macrophages is context-dependent, with potential pro-tumorigenic outcomes observed in certain cancer models. Many studies have highlighted the importance of Notch signalling in controlling the phenotypic switching of macrophages within tumours, exerting the balance between pro- and anti-tumour immune responses. The activation of Notch, particularly Notch1/2 in CD8^+^ T cells, stimulates a robust anti-tumour response. It has assorted functionalities in the stimulation of effector T cells in an immunosuppressive tumour microenvironment and promotes tumour clearance via cytolytic mediators like granzyme B and IFN-γ in mouse models. This proposes that targeting the Notch cascade in CD8^+^ T cells could serve as a promising strategy for boosting anti-tumour immunity. However, chronic Notch stimulation via APC contributes to PD-1-mediated T-cell exhaustion. Targeted management of the Notch pathway may aid in overcoming MDSC-arbitrated immunosuppression and improving T-cell efficacy, signifying possible therapeutic openings in oncotherapy. Although the expression of Notch1 in BCSCs is suspected to be inversely related to T cell-mediated cytotoxicity, the role of Notch in epithelial–mesenchymal transition (EMT) via CSC–T cell interaction is substantially dicey and needs further experimental validations for better understanding. Patient data regarding the activity of Notch-mediated T-cell response are scarce. Several therapeutic strategies have been developed based on genetic signatures specific to tumour cells in varying kinds of malignancies. There is a sufficient lacuna in the characterization of intratumoral T cells in patients. Even then, an array of Notch-targeted T cell-mediated therapeutic regimes have been experimentally validated as discussed previously. GSIs, CAR T cells, and Notch-associated oncolytic virotherapy have obtained considerable success in preclinical experimental outcomes and initial phases of clinical trials, which abets the first-line use of tumour-specific, economic, and safe Notch therapeutics in patient care. SynNotch CAR T cells are especially fascinating, as their targeted cytotoxic response provides an improved and explicit anti-tumour response. Apart from the assessments of risk and efficiency of Notch therapeutics, it should also be ensured that persistent Notch activation does not result in leukemic mutations of T cells or advanced tumour progression. Notch signalling serves as an oncogenic driver in various malignancies like T-cell acute lymphoblastic leukaemia/lymphomas and peripheral T-cell lymphomas, which complicates its role in cancer biology. While Notch signalling holds a promising therapeutic target for enhancing anti-tumour immunity, its subtle effects on immune cell function and tumour progression should be carefully considered for better clinical translation. In the era of precision medicine, emerging experimental evidence in specific malignancies is aiding substantial progress in the domain of Notch-mediated remedies in T cell-based targeted oncotherapy.

## Glossary

ACT: adoptive T-cell therapy
ADAM: protein family (“A disintegrin and metalloprotease”)
ANK: ankyrin repeat
APC: antigen-presenting cells
BCR: B cell receptor
CAR: chimeric antigen receptor
CCR7: C-C chemokine receptor type 7
CCL2: C-C chemokine ligand 2
CD4+: cluster of differentiation 4+
CD8+: cluster of differentiation 8+
cMYC: cellular myelocytomatosis oncogene
CSC: cancer stem cell
CTL: cytotoxic T lymphocytes
CTLA4: cytotoxic T-cell lymphocyte-associated protein 4
DAPT: N-[N-(3,5-difluorophenacetyl)-L-alanyl]-S-phenylglycine t-butyl ester
DDIS: DNA Damage-Induced Senescence
DLL: Delta-like ligand
EGF: epidermal growth factor
EGFR: epidermal growth factor receptor
EMT: epithelial–mesenchymal transition
EOMES: eomesodermin
EOC: epithelial ovarian cancer
ER: estrogen receptor
ERK: extracellular signal-regulated kinase
EZH2: enhancer of zeste homolog 2
FOXA2: forkhead box protein A2
GATA3: GATA binding protein 3
GATα: GGA and Tom1 domain
GEP: gene expression profiling
GSI: gamma secretase inhibitors
hes: Hairy enhancer of split
hey: Hairy related
HES4: Hes family BHLH transcription factor 4
HNSCC: head and neck squamous cell carcinoma
ICB: immune checkpoint blockade
ICI: immune checkpoint inhibitor
IFN-γ: interferon gamma
IL-6/8: interleukin-6/8
iPSCs: induced pluripotent stem cells
iTSCM: induced T memory stem cells
JAG: Jagged1
KLK6: Kallikrein-related peptidase 6
KLRG: Killer cell lectin-like receptor subfamily G member 1
LLC: Lewis lung carcinoma
LSD1: demethylase 1a
MAML: Mastermind-like
MDSCs: myeloid-derived suppressor cells
MET: mesenchymal–epithelial transition
MHC-II: major histocompatibility complex II
MMP9: matrix metalloproteinase 9
MPEC: Memory Precursor and Short-Lived Effector cells
MRK 560: γ-secretase inhibitor
MYC: myelocytomatosis oncogene
NED: Notch extracellular domain
NF-I: neurofibromatosis type 1
NF-κB: nuclear factor kappa B
NICD: Notch intracellular cytoplasmic domain
NK: natural killer
NLS: nuclear localization signal
NPC: neural precursor cells
NRR: negative regulatory region
NSCLC: non-small cell lung cancer
oHSV: oncolytic herpes simplex virus
OXPhos: oxidative phosphorylation
PD-L1: programmed death ligand 1
PD-1: programmed cell death protein 1
PECAM: platelet endothelial cell adhesion molecule
PEST: a polypeptide sequence rich in proline (P), glutamic acid (E), serine (S), and threonine (T)
PI3K/AKT/mTORC1 cascade: phosphoinositide-3-kinase/protein kinase B/mammalian target of rapamycin complex 1
PTCL: peripheral T-cell lymphoma
RBPJ: recombination signal binding protein for immunoglobulin kappa J region
RIS: Replicative-Induced Senescence
RTK: receptor tyrosine kinase
SASP: senescence-associated secretory phenotype
SLEC: short-lived effector cells
STAT3: signal transducer and activator of transcription 3
T ALL: T-cell acute lymphoblastic leukaemia/lymphomas
TAM: tumour-associated macrophage
TBX2: T-box transcription factor 2
TCIM: traditional complementary and integrative medicine
TCR: T-cell receptor
TNBC: Triple-negative breast cancer
TGF-β: transforming growth factor beta
TIGIT: T-cell immunoreceptor with Ig and ITIM domains
TIL: tumour-infiltrating lymphocytes
TIM3: T-cell immunoglobulin and mucin domain 3
TMB: tumour mutational burden
TME: tumour microenvironment
TNFα: tumour necrosis factor alpha
TRAIL: tumour necrosis factor-related apoptosis-inducing ligand
VEGF: vascular endothelial growth factor
